# An overview of eight- and nine-coordinate *N*-donor solvated lanthanoid(III) and actinoid(III) ions

**DOI:** 10.1007/s10967-018-5757-9

**Published:** 2018-02-21

**Authors:** Daniel Lundberg

**Affiliations:** Department of Molecular Sciences, Uppsala BioCenter, Swedish University of Agricultural Sciences, P.O. Box 7015, 750 07 Uppsala, Sweden

**Keywords:** Lanthanoid(III) ions, Actinoid(III) ions, *N*-donor ligands, Solvation, Separation and transmutation, Coordination chemistry

## Abstract

The use of replacement lanthanoid ions in actinoid chemistry is commonplace, which requires a full understanding of the similarities and differences between the two series. This overview lists, compares and discusses the available crystallographic data for *N*-donors for the lanthanoids and the actinoids using their trivalent state as a natural starting point for comparison.

**Electronic supplementary material:**

The online version of this article (10.1007/s10967-018-5757-9) contains supplementary material, which is available to authorized users.

## Introduction

After the initial use in the nuclear fuel cycle, untreated spent nuclear waste (SNW) requires very long storage times in bedrock repositories typically in the order of 100,000 years [1, 2]. Using modern solvent extraction methods, however, the storage can be shortened by several orders of magnitude, perhaps down to 100 years, by separating and transmutating the most highly radioactive isotopes, the minor actinoids, in the SNW [3–5]. Several different modern solvent extraction techniques exist already [6–10], and novel ones are continuously being developed to make sure that this type of treatment becomes a reality on a large scale. To understand the extraction techniques, it is common to work with replacement ions, usually lanthanoid ions, in the early stages of development before moving on to the highly radioactive material. This contribution is meant to work as a guideline for this kind of research focusing on the similar ionic radii of the metal ions involved, in addition to being a comparative study of said ions.

## Theory

For the actinoid(III) ions the most common replacement is one or more of the lanthanoid(III) ions, due to their similarity in both size and chemistry. It is important to point out that while there are differences between the two series, for instance the possible oxidation states, the sizes of their trivalent ions is their dominant similarity hence this overview focuses on this fact. The relative sizes of the ions in both series were recently compared using *O*-donor ligands [11], proving from a structural point-of-view that there is no evidence why the commonly used europium(III) ion should be used as a replacement for the americium(III) ion. Instead, the neodymium(III) ion is a much better candidate to simulate the size of americium(III), while europium(III) instead ought to work well as a replacement for einsteinium(III). With this new set of actinoid(III) radii [11], which on a statistical basis greatly improves the ones offered by Shannon [12], it is possible to draw appropriate conclusions in separating ions from one another based on their actual size. While older separation methods used *O*-donors, many of the new and effective complexation ligands are *N*-donors, which is why this overview of published results compares the *N*-donor solvated lanthanoid(III) series with those of the actinoid(III) ions.

## Experimental

Using the two largest crystallographic databases available, the Cambridge Structural Database (CSD) [13] and the Inorganic Crystal Structure Database (ICSD) [14], all eight- and nine-coordinate lanthanoid(III) and actinoid(III) ions with *N*-donors were compiled and compared on the basis of their bond distance, Table S1, using the ionic radii previously published [11, 15]. Although available, data sets for the lanthanoid(III) and actinoid(III) ions obtained from experiments in solution are not included, due to the difficulty of accurately determining such bond lengths and coordination numbers; instead this study, whenever needed, includes available data for the divalent and tetravalent states of the lanthanoids in the solid state.

## Results and discussion

**Fig. 1:**
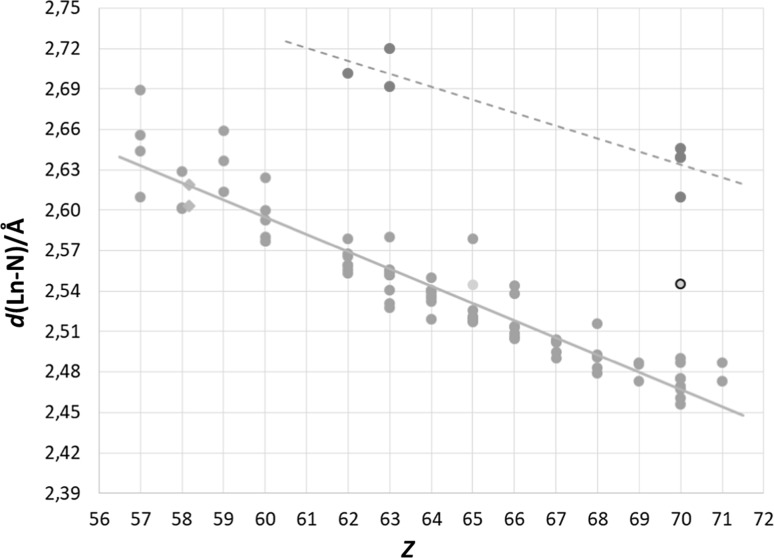
All selected neutral, eight-coordinate *N*-donor lanthanoid(III) (green circles) structures found in the database search. The two uranium(III) structures (blue diamonds) have been placed on uranium’s fractional lanthanoid atomic number, *Z*_Ln_ = 58.17, as described in ref. 11. The marked ytterbium(III) structure (yellow circle with black line) is likely a terbium(III) one (yellow circle without line). As seen here, the seven reported divalent structures (red circles) have significantly different mean Ln–N bond distances, *d*(Ln-N)

The full database search included over 400 eight-coordinate *N*-donor lanthanoid and actinoid structures, Table S1, though several of these were divalent and tetravalent structures. Similarly, for the nine-coordinate complexes, there were over 160 reported lanthanoid and actinoid structures, Table S2. There is no doubt about that the contraction of the respective series are close to being parallel to the one found for *O*-donors, even though there is a very limited amount of data for the actinoid(III) series. The fact that nitrogen is described as a softer ligand atom in hard and soft acids and bases (HSAB) theory [16] also becomes obvious since the mean Ln–N bond distances show a much greater distribution than the mean Ln–O bond distance distribution of *O*-donors. However, for various reasons, not all complexes are suitable for a bond length comparison, meaning that those with both partially and fully anionic ligands with Coulombic interactions, structures with ligand molecules restricting the bonding geometry, and close bidentate ligands are excluded [11]. Also, given the unusual triple bond of acetonitrile, a small number of such complexes have been excluded. After this approach, approximately 80 eight-coordinate lanthanoid(III) and two uranium(III) structures remain, Fig. 1 and Table S1. The remainder of this overview will be based on these selected structures, with the corresponding treatment for the nine-coordinate species.

### Eight-coordinate *N*-donor lanthanoid(III) and actinoid(III) structures

The mean Ln–N bond length distribution for each element is ± 0.06 Å, compared to ± 0.03 Å in the corresponding case for *O*-donors [11, 15]. This is an effect of the softer donor capabilities of the nitrogen atom, though the actual “softness” is heavily dependent on the bonding situation for the nitrogen atom in question. While the full data search included all types of *N*-donors, a better overview is obtained if one focuses on a few selected homoleptic *N*-donor “families”, namely: diammines, tris(benzimidazolylmethyl)amines (TBTA), and tris(2-pyridylmethyl)amines (TPMA). All the selected families cover a large part of the lanthanoid (and thus actinoid) series, and are as such of interest not only in regards to the mentioned separation techniques, but also to chemists in general to indicate how different types of *N*-donors behave.

#### Diammines (bidentate)

**Fig. 2:**
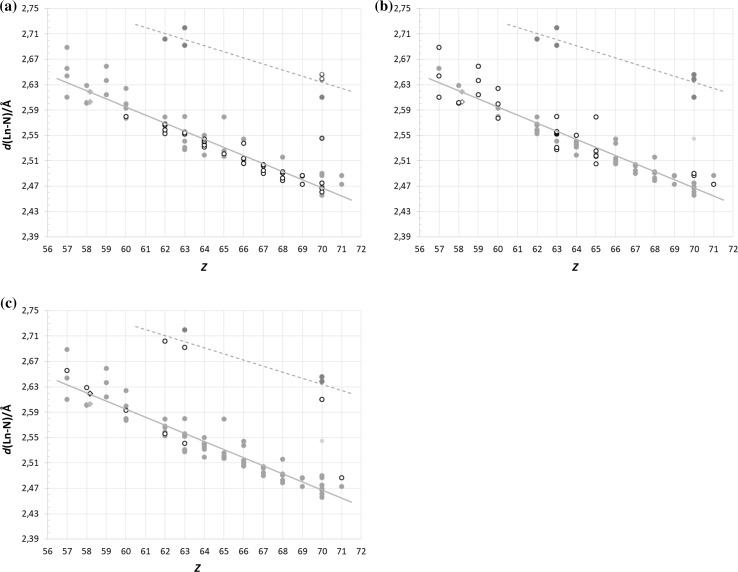
All selected neutral, eight-coordinate *N*-donor lanthanoid(III) (green circles), lanthanoid(II) (red circles), and actinoid(III) (blue diamonds) structures in the data search with the respective families highlighted (white symbols with black outline): (**a**) ammines, (**b**) tris(benzimidazolylmethyl)amines, and (**c**) tris(2-pyridylmethyl)amines. The bidentately-binding diammine ytterbium(III) structure (yellow circle) is likely a terbium(III) one. The highlighted uranium(III) structures (white diamonds) fits nicely with the corresponding structures when placed on its fractional lanthanoid atomic number, *Z*_Ln_ = 58.17, as described in ref. 11

There are over 30 different diammine lanthanoid(III) structures which have mean Ln–N bond distances spread fairly evenly around the trendline for all neutral *N*-donors, Fig. 2a. The mean Ln–N bond distance is of medium size and also exhibits a very narrow bond distribution making diammines, essentially, the standard of *N*-donors with an expected “normal” nitrogen radius. With the help of this fact, one can even say that one reported ytterbium(III) structure [17], most likely, is a terbium(III) one, further supported by the very similar names of these two elements in most languages. On the basis of chemical similarity, a monodentate diammine gadolinium(III), an octaammine ytterbium(III), and two octaammine ytterbium(II) structures were also added to this family.

#### Tris(benzimidazolylmethyl)amine (tetradentate)

There are almost 30 different tris(benzimidazolylmethyl)amine lanthanoid(III) structures (white circles) spread across the entire lanthanoid series, and also one of the uranium(III) structures (white diamond), Fig. 2b. Their mean Ln–N bond distance distribution within each element is quite large, in fact, this ligand family is the reason for the spread of the neutral *N*-donors. Nevertheless, on average, they have the same expected mean Ln–N bond distance as any neutral *N*-donor.

#### Tris(2-pyridylmethyl)amines (tetradentate)

There are significantly fewer tris(2-pyridylmethyl)amine lanthanoid(III) structures, but they give valuable insight to the divalent state of the lanthanoids, Fig. 2c. They seemingly have a narrower bond distribution than the tris(benzimidazolylmethyl)amine structures, but this could be an effect of the limited number of structures available. The three divalent lanthanoid structures yield a lanthanoid(II) radius approximately 0.15 Å longer than their trivalent counterpart. The sole actinoid(III) structure is once again a uranium(III) one, which follows the expected pattern when placed on its fractional lanthanoid atomic number, *Z*_Ln_ = 58.17 [11].

### Nine-coordinate *N*-donor lanthanoid(III) and actinoid(III) structures

**Fig. 3:**
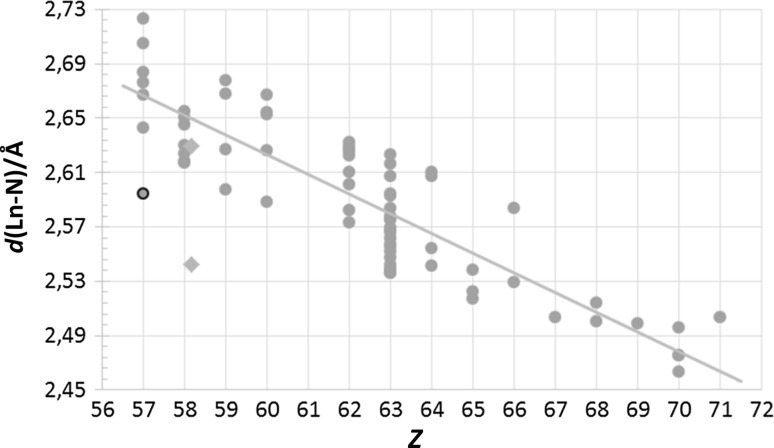
All selected neutral, nine-coordinate *N*-donor lanthanoid(III) (green circles) structures found in the database search. The uranium(III) structures (blue diamonds) have been placed on uranium’s fractional lanthanoid atomic number, *Z*_Ln_ = 58.17, as described in ref. 11. One structure (black outline) was treated as an outlier and excluded from the calculation of the mean Ln–N bond distance, *d*(Ln-N). One divalent nine-coordinate europium(II) structure, reported as trivalent, lies outside the shown range at a mean Ln–N bond distance of *d*(Ln-N) = 2.808 Å

The exclusion principles for nine-coordinate structures follow the same pattern as for the eight-coordinate species. Thus, after sorting out non-trivalent structures, dinuclear species, those with both partially and fully anionic ligands, ligand molecules with restricting bonding geometry, close bidentate ligands, and acetonitrile complexes, 73 nine-coordinate lanthanoid(III) and two uranium(III) structures remain, Fig. 3. It is quite obvious that europium has been favoured for *N*-donor studies, as 30 per cent of the structures contain the element. This is, at least in part, due to the incorrect assumption that the europium(III) ion is a suitable replacement for the americium(III) ion [11]. The mean Ln–N bond distance spread within each element in nine-coordination is nearly the same as eight-coordinate *N*-donor species, ± 0.05 Å. The lanthanoid contraction is also clearly visible, but to evaluate the different types of *N*-donors a closer look is needed, which here is represented by three tridentate *N*-donor ligand families: diethylenetriamines, 2,2′:6′,2″-terpyridines (Terpy), and 2,6-bis(1,2,4-triazinyl)pyridines (BTP).

#### Diethylenetriamine (tridentate)

**Fig. 4:**
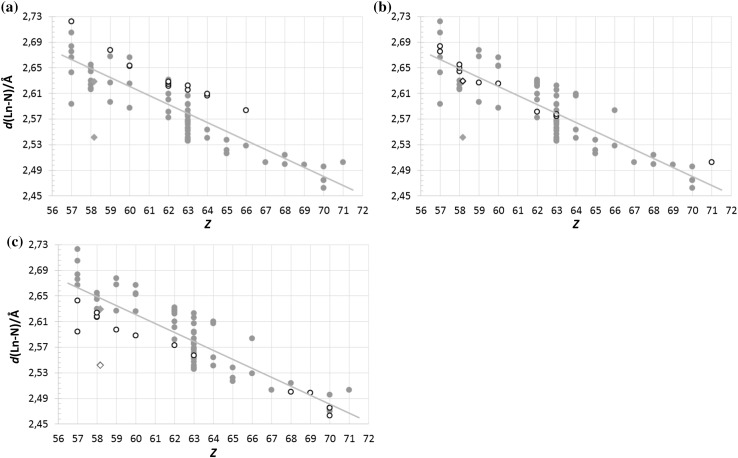
All selected neutral, nine-coordinate *N*-donor lanthanoid(III) (green circles) and actinoid(III) (blue diamonds) structures in the data search with the respective families highlighted (white symbols with black outline): (**a**) diethylenetriamines, (**b**) 2,2′:6′,2″-terpyridines, and (**c**) 2,6-bis(1,2,4-triazinyl)pyridines. It becomes obvious that the two uranium structures (white diamonds) have different oxidation states, where the 2,2′:6′,2″-terpyridine structure in (**b**) is trivalent and the 2,6-bis(1,2,4-triazinyl)pyridine one in (**c**) is tetravalent (although reported as trivalent)

Even though the thirteen reported structures only cover lanthanum(III) to dysprosium(III), the diethylenetriamine solvates have among the longest nine-coordinate mean Ln–N bond distances, when compared to all neutral nine-coordinate *N*-donor structures, Fig. 4a. The radius of this type of *N*-donor nitrogen atom is thus larger than the average trendline value. The bond distance spread within the family is small, perhaps due to the limited amount of data available.

#### 2,2′:6′,2″-terpyridines (tridentate)

The 2,2′:6′,2″-terpyridine lanthanoid(III) and the uranium(III) structures have a bit larger spread, but exhibit a mean value around the trendline value, Fig. 4b, following the average nitrogen radius quite well. The mean Lu-N bond distance of the lutetium(III) structure is slightly increased and off the trendline, perhaps due to crowding effects similar to those seen in the hydrated lanthanoid(III) complexes [18].

#### 2,6-bis(1,2,4-triazinyl)pyridines (tridentate)

The shorter mean Ln–N bond distance of 2,6-bis(1,2,4-triazinyl)pyridine lanthanoid(III) structures is more pronounced early in the lanthanoid series, but visible until the end, Fig. 4c. The lanthanum(III) structure with the shorter La–N bond distance deviates more than expected and was not included in the calculation of the overall Ln–N bond distance slope. Furthermore, the 2,6-bis(1,2,4-triazinyl)pyridine uranium structure [19] is not trivalent as reported, but rather tetravalent which explains the significantly shorter distance than expected for an actual trivalent species.

### Comparison of eight- and nine-coordinate *N*-donor lanthanoid(III)/actinoid(III) complexes

**Fig. 5:**
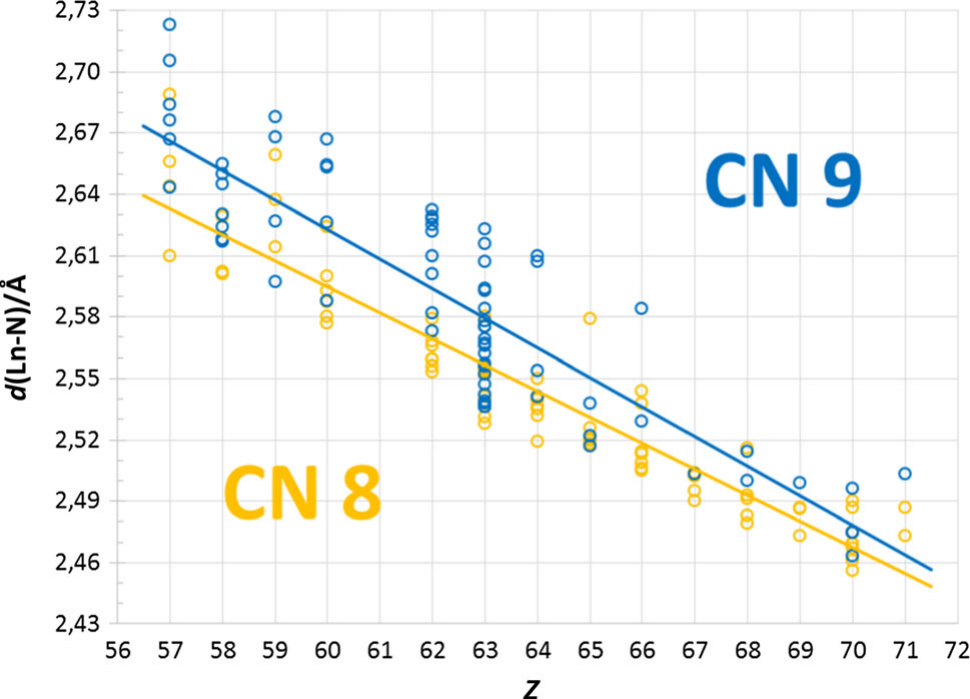
All selected eight- and nine-coordinate *N*-donor lanthanoid(III) structures in the present study (orange and blue circles, respectively). The overlap between the coordination numbers stem from the softness of nitrogen as a donor atom, which offers greater variability than that of a harder ligand atom, e.g. oxygen

The main difference between eight- and nine-coordinate *N*-donor complexes is the number of coordinated ligand molecules, where the two prevalent denticites in eight-coordination is bidentate and tetradentate, while the nine-coordinate complexes feature almost exclusively tridentante ligands. This is in part a result of the exclusion of certain structures, but given the possibility of strained coordination geometries one to be preferred. Unlike the study with *O*-donors complexes [11], and given the fairly large mean Ln–N bond distance spread that both these coordination numbers feature, it is not surprising that they overlap to a certain extent when plotted together, Fig. 5.

This rather severe overlap does present a problem when trying to establish coordination numbers in solution, where the main guiding information is a mean bond distance. Normally, there is also a good chance of knowing which ligand is present in that solution which, through its denticity, will give additional information regarding the correct structure. Care should be taken, however, since there is no guarantee that the coordination number and geometry found in the solid state is the prevalent one in solution. One prime example of this is the solvation of a *bis*-triazinyl bipyridine (BTBP) [20, 21] and europium(III) nitrate in various organic solvents [22]. In solution, the europium(III) ion coordinates two BTBP ligands for a coordination number of eight, whereas a crystal structure precipitating from one of these solutions also includes a bidentately bound nitrate ion, making it effectively ten-coordinate [22]. Furthermore, when americium(III) was treated in the same way, it was found that the americium(III) ion thanks to its larger size coordinates a nitrate even in solution, and was determined to be at least nine-coordinate [23].

## Conclusions

This overview is meant to assist in the determination of coordination numbers in cases where *N*-donors coordinate to lanthanoid(III) and actinoid(III) ions. The selection of ligands have been carefully performed in such a way to minimize errors due to charge effects and strained coordination geometries. That said, one should keep in mind the limited amount of crystallographic data that exist for the actinoid(III) ions and their possibility to alter oxidation state. Through this method, it has been possible to detect a few errors in reported valency, a possible mislabelled chemical structure, but also the different sizes of various types of *N*-donor ligand nitrogens through the use of previously presented ionic radii.

## Electronic supplementary material

Below is the link to the electronic supplementary material.

Supplementary material 1 (DOC 813 kb)
