# Supplementary material for: An overview of eight- and nine-coordinate N-donor solvated lanthanoid(III) and actinoid(III) ions
Source: J Radioanal Nucl Chem. 2018 Feb 21;316(2):849–54. doi: 10.1007/s10967-018-5757-9 (PMC5920002; doi:10.1007/s10967-018-5757-9)
Supplement: Supplementary file 1 — Supplementary material 1 (DOC 813 kb) [file 10967_2018_5757_MOESM1_ESM.doc]

Supplementary information

**Table S1** All eight-coordinate trivalent (left column set), tetravalent (middle column set), and divalent (right column set) *N*-donor lanthanoid and actinoid structures reported in Cambridge Structural Database (CSD). The structures are ordered after atomic number, *Z*, mean Ln-N bond distance, and CSD retrieval code. Structures in red typeface were excluded in the final analysis for the given reason.

| **Z** | ***d*(Ln-N)** | **CSD code** | **Z** | ***d*(Ln-N)** | **CSD code** | **Z** | ***d*(Ln-N)** | **CSD code** | **reason for exclusion** |
| --- | --- | --- | --- | --- | --- | --- | --- | --- | --- |
| *trivalent structures* | | | *tetravalent structures* | | | *divalent structures* | | |  |
| 57 | 2.513 | ZEHTUB |  |  |  |  |  |  | constrained |
| 57 | 2.536 | QOCJUN |  |  |  |  |  |  | constrained |
| 57 | 2.553 | BASKOW |  |  |  |  |  |  | constrained |
| 57 | 2.554 | YEGTIN |  |  |  |  |  |  | constrained |
| 57 | 2.555 | EXIMIH |  |  |  |  |  |  | close bidentate |
| 57 | 2.555 | QOCJOH |  |  |  |  |  |  | constrained |
| 57 | 2.556 | ZAZQAS |  |  |  |  |  |  | constrained |
| 57 | 2.559 | TUPWEG |  |  |  |  |  |  | constrained |
| 57 | 2.571 | QOMMOU |  |  |  |  |  |  | close bidentate |
| 57 | 2.574 | EXILOM |  |  |  |  |  |  | close bidentate |
| 57 | 2.577 | JEKVEA |  |  |  |  |  |  | anionic ligands |
| 57 | 2.577 | JEKXEC |  |  |  |  |  |  | anionic ligands |
| 57 | 2.610 | FETQIE |  |  |  |  |  |  |  |
| 57 | 2.618 | EWOMOU |  |  |  |  |  |  | constrained |
| 57 | 2.623 | AHOJUD |  |  |  |  |  |  |  |
| 57 | 2.625 | TEJYOX |  |  |  |  |  |  |  |
| 57 | 2.635 | JAMPOD |  |  |  |  |  |  | close bidentate |
| 57 | 2.636 | LUZCIS |  |  |  |  |  |  | constrained |
| 57 | 2.641 | WOSSII |  |  |  |  |  |  | constrained |
| 57 | 2.644 | HAZFET |  |  |  |  |  |  |  |
| 57 | 2.656 | LARGER |  |  |  |  |  |  |  |
| 57 | 2.665 | AXADIM |  |  |  |  |  |  | anionic ligands |
| 57 | 2.689 | WONSOI |  |  |  |  |  |  |  |
|  |  |  | 58 | 2.349 | MAVTOV |  |  |  | +4, anionic ligands |
|  |  |  | 58 | 2.386 | ZUVWIY |  |  |  | +4, anionic ligands |
|  |  |  | 58 | 2.412 | UVEHEK |  |  |  | +4, constrained |
|  |  |  | 58 | 2.415 | JONZAN |  |  |  | +4, constrained |
|  |  |  | 58 | 2.419 | ICISIW |  |  |  | +4, constrained |
|  |  |  | 58 | 2.422 | VIGRUZ |  |  |  | +4, constrained |
|  |  |  | 58 | 2.429 | UVEHAG |  |  |  | +4, constrained |
|  |  |  | 58 | 2.43 | ZUVWEU |  |  |  | +4, constrained |
|  |  |  | 58 | 2.439 | RABDEZ |  |  |  | +4, constrained |
|  |  |  | 58 | 2.439 | SACNUF |  |  |  | +4, constrained |
|  |  |  | 58 | 2.446 | NIMZEQ |  |  |  | +4, constrained |
|  |  |  | 58 | 2.447 | QIVJIQ |  |  |  | +4, constrained |
|  |  |  | 58 | 2.449 | SACNOZ |  |  |  | +4, constrained |
|  |  |  | 58 | 2.452 | PEJNAU |  |  |  | +4, constrained |
|  |  |  | 58 | 2.458 | ALIZOK |  |  |  | +4, constrained |
|  |  |  | 58 | 2.46 | ALIZIE |  |  |  | +4, constrained |
|  |  |  | 58 | 2.475 | DURLUX |  |  |  | +4, constrained |
|  |  |  | 58 | 2.476 | VEGXEK |  |  |  | +4, constrained |
| 58 | 2.483 | EZOYEZ |  |  |  |  |  |  | constrained |
| 58 | 2.486 | UVEHOU |  |  |  |  |  |  | constrained |
| 58 | 2.488 | EZOYAV |  |  |  |  |  |  | constrained |
| 58 | 2.503 | ZUVWOE |  |  |  |  |  |  | close bidentate |
| 58 | 2.534 | QOCJIB |  |  |  |  |  |  | constrained |
| 58 | 2.548 | JEKVIE |  |  |  |  |  |  | anionic ligands |
| 58 | 2.551 | NIMZAM |  |  |  |  |  |  | constrained |
| 58 | 2.570 | JUHTAH |  |  |  |  |  |  | constrained |
| 58 | 2.580 | VONMER |  |  |  |  |  |  | constrained |
| 58 | 2.593 | ETOZIY |  |  |  |  |  |  | anionic ligands |
| 58 | 2.601 | CILTUN |  |  |  |  |  |  |  |
| 58 | 2.601 | JUDPAZ |  |  |  |  |  |  | constrained |
| 58 | 2.602 | CILVAV |  |  |  |  |  |  |  |
| 58 | 2.604 | LEFVEY |  |  |  |  |  |  |  |
| 58 | 2.608 | WOSSAA |  |  |  |  |  |  | constrained |
| 58 | 2.618 | TANMUQ |  |  |  |  |  |  | constrained |
| 58 | 2.629 | LARGIV |  |  |  |  |  |  |  |
| 58 | 2.630 | DOSYOZ10 |  |  |  |  |  |  | constrained |
| 58 | 2.648 | QIVKIR |  |  |  |  |  |  | constrained |
|  |  |  | 59 | 2.476 | KOBRUO |  |  |  | +4, constrained |
| 59 | 2.492 | QOCKEY |  |  |  |  |  |  | constrained |
| 59 | 2.494 | QOCLAV |  |  |  |  |  |  | constrained |
| 59 | 2.495 | WAYSIY |  |  |  |  |  |  | constrained |
| 59 | 2.506 | CINPEW |  |  |  |  |  |  | constrained |
| 59 | 2.514 | BASKUC |  |  |  |  |  |  | constrained |
| 59 | 2.535 | JEKVOK |  |  |  |  |  |  | constrained |
| 59 | 2.559 | SAMVUX01 |  |  |  |  |  |  | constrained |
| 59 | 2.560 | SAMVUX |  |  |  |  |  |  | constrained |
| 59 | 2.614 | JOSTAM |  |  |  |  |  |  |  |
| 59 | 2.637 | WONSUO01 |  |  |  |  |  |  |  |
| 59 | 2.659 | WONSUO |  |  |  |  |  |  |  |
| 60 | 2.465 | CIZGIB06 |  |  |  |  |  |  | constrained |
| 60 | 2.466 | IWARAZ |  |  |  |  |  |  | constrained |
| 60 | 2.471 | CIZGIB02 |  |  |  |  |  |  | constrained |
| 60 | 2.471 | JOBFUD |  |  |  |  |  |  | constrained |
| 60 | 2.493 | CIZGIB07 |  |  |  |  |  |  | constrained |
| 60 | 2.497 | EXIMON |  |  |  |  |  |  | close bidentate |
| 60 | 2.501 | CINPIA |  |  |  |  |  |  | constrained |
| 60 | 2.505 | BASLAJ |  |  |  |  |  |  | constrained |
| 60 | 2.514 | EXILUS |  |  |  |  |  |  | constrained |
| 60 | 2.516 | NAKXUS |  |  |  |  |  |  | constrained |
| 60 | 2.518 | KEFDEE01 |  |  |  |  |  |  | close bidentate |
| 60 | 2.522 | JEKWAX |  |  |  |  |  |  | anionic ligands |
| 60 | 2.523 | LUDQIK |  |  |  |  |  |  | close bidentate |
| 60 | 2.552 | TUCTOB |  |  |  |  |  |  | constrained |
| 60 | 2.552 | XAKYIT |  |  |  |  |  |  |  |
| 60 | 2.556 | DULBIV |  |  |  |  |  |  | constrained |
| 60 | 2.556 | XAKYAL |  |  |  |  |  |  |  |
| 60 | 2.561 | XAKXOY |  |  |  |  |  |  |  |
| 60 | 2.564 | LEFVOI |  |  |  |  |  |  |  |
| 60 | 2.565 | POZLIZ |  |  |  |  |  |  | constrained |
| 60 | 2.570 | UFADUA |  |  |  |  |  |  | constrained |
| 60 | 2.573 | WUGBOP |  |  |  |  |  |  | constrained |
| 60 | 2.577 | EWOMIO |  |  |  |  |  |  | constrained |
| 60 | 2.577 | HAZFIX |  |  |  |  |  |  |  |
| 60 | 2.580 | ZETTOJ |  |  |  |  |  |  |  |
| 60 | 2.584 | WOSTEF |  |  |  |  |  |  | constrained |
| 60 | 2.587 | NAVCIW |  |  |  |  |  |  | acetonitrile |
| 60 | 2.593 | LARGOB |  |  |  |  |  |  |  |
| 60 | 2.600 | WESSOC |  |  |  |  |  |  |  |
| 60 | 2.624 | WESSES |  |  |  |  |  |  |  |
| 62 | 2.436 | GUCNUO |  |  |  |  |  |  | close bidentate |
| 62 | 2.455 | QOCKIC |  |  |  |  |  |  | constrained |
| 62 | 2.455 | VAJKAT |  |  |  |  |  |  | constrained |
| 62 | 2.456 | VAJKAT01 |  |  |  |  |  |  | constrained |
| 62 | 2.457 | ZOMWUT |  |  |  |  |  |  | constrained |
| 62 | 2.458 | VEFBAK |  |  |  |  |  |  | constrained |
| 62 | 2.459 | CINPAS |  |  |  |  |  |  | constrained |
| 62 | 2.469 | OBELOX |  |  |  |  |  |  | constrained |
| 62 | 2.475 | JEKWEB |  |  |  |  |  |  | anionic ligands |
| 62 | 2.486 | UFADAG |  |  |  |  |  |  | constrained |
| 62 | 2.496 | ACIXOA |  |  |  |  |  |  | constrained |
| 62 | 2.500 | ACIXEQ |  |  |  |  |  |  | constrained |
| 62 | 2.503 | GOTCIB |  |  |  |  |  |  | constrained |
| 62 | 2.504 | JIQVEL |  |  |  |  |  |  | constrained |
| 62 | 2.513 | XAKYEP |  |  |  |  |  |  |  |
| 62 | 2.519 | XUSNII |  |  |  |  |  |  |  |
| 62 | 2.527 | WOSSEE |  |  |  |  |  |  | constrained |
| 62 | 2.528 | LEFVIC |  |  |  |  |  |  |  |
| 62 | 2.532 | WIVCIO |  |  |  |  |  |  | anionic ligands |
| 62 | 2.534 | OCOCAK |  |  |  |  |  |  | close bidentate |
| 62 | 2.550 | TABDUW |  |  |  |  |  |  | constrained |
| 62 | 2.551 | YUBPAM |  |  |  |  |  |  | constrained |
| 62 | 2.553 | GAKLIP |  |  |  |  |  |  |  |
| 62 | 2.556 | LUPHOV |  |  |  |  |  |  |  |
| 62 | 2.558 | EWOMEK |  |  |  |  |  |  | constrained |
| 62 | 2.558 | WOSVAD |  |  |  |  |  |  | constrained |
| 62 | 2.558 | WOSVEH |  |  |  |  |  |  | constrained |
| 62 | 2.559 | GABJEB |  |  |  |  |  |  |  |
| 62 | 2.559 | JAJQIV |  |  |  |  |  |  |  |
| 62 | 2.566 | FISPUT |  |  |  |  |  |  |  |
| 62 | 2.568 | NEMSEE |  |  |  |  |  |  |  |
| 62 | 2.576 | XEPLAF |  |  |  |  |  |  |  |
| 62 | 2.578 | ILEDAG |  |  |  |  |  |  | close bidentate |
| 62 | 2.579 | WIGVEN |  |  |  |  |  |  |  |
|  |  |  |  |  |  | 62 | 2.607 | ZOMXAA | constrained, +2/+3? |
|  |  |  |  |  |  | 62 | 2.702 | LUPHIP | ok, +2 |
| 63 | 2.432 | TIQNUE |  |  |  |  |  |  | constrained |
| 63 | 2.432 | VAJKEX01 |  |  |  |  |  |  | constrained |
| 63 | 2.433 | VAJKEX |  |  |  |  |  |  | constrained |
| 63 | 2.434 | CEHKOQ |  |  |  |  |  |  | constrained |
| 63 | 2.437 | JOBGAK |  |  |  |  |  |  | constrained |
| 63 | 2.439 | VIGQUY |  |  |  |  |  |  | constrained |
| 63 | 2.442 | ZUNJID |  |  |  |  |  |  | constrained |
| 63 | 2.459 | OCOBIR |  |  |  |  |  |  | constrained |
| 63 | 2.460 | IMAQAP |  |  |  |  |  |  | constrained |
| 63 | 2.467 | XUXCOJ01 |  |  |  |  |  |  | constrained |
| 63 | 2.471 | FEFBUP |  |  |  |  |  |  | constrained |
| 63 | 2.471 | XUXCOJ |  |  |  |  |  |  | constrained |
| 63 | 2.474 | JEKWIF |  |  |  |  |  |  | anionic ligand |
| 63 | 2.476 | ACIXIU |  |  |  |  |  |  | constrained |
| 63 | 2.490 | HOKQOP |  |  |  |  |  |  | constrained |
| 63 | 2.490 | GAWCAL |  |  |  |  |  |  | constrained |
| 63 | 2.492 | TIQNOY |  |  |  |  |  |  | constrained |
| 63 | 2.494 | EFIZIE |  |  |  |  |  |  | constrained |
| 63 | 2.495 | ACIXUG |  |  |  |  |  |  | constrained |
| 63 | 2.498 | QUSFER |  |  |  |  |  |  | constrained |
| 63 | 2.502 | DAFJAY |  |  |  |  |  |  | anionic ligand |
| 63 | 2.509 | VIGRAF |  |  |  |  |  |  | constrained |
| 63 | 2.510 | GAPRUK |  |  |  |  |  |  | constrained |
| 63 | 2.521 | TUCTIV |  |  |  |  |  |  | constrained |
| 63 | 2.522 | ITEDUH |  |  |  |  |  |  | constrained |
| 63 | 2.524 | TOVPAX |  |  |  |  |  |  | constrained |
| 63 | 2.524 | GAVXUZ |  |  |  |  |  |  | constrained |
| 63 | 2.528 | XOSVAD |  |  |  |  |  |  |  |
| 63 | 2.530 | EHEYEW |  |  |  |  |  |  | constrained |
| 63 | 2.531 | FATKUH |  |  |  |  |  |  | constrained |
| 63 | 2.531 | FAWWUV |  |  |  |  |  |  |  |
| 63 | 2.541 | LARHAO |  |  |  |  |  |  |  |
| 63 | 2.543 | TABFAE |  |  |  |  |  |  | constrained |
| 63 | 2.552 | JOSQUD |  |  |  |  |  |  |  |
| 63 | 2.552 | RAYBUP |  |  |  |  |  |  |  |
| 63 | 2.553 | HAZGAQ |  |  |  |  |  |  |  |
| 63 | 2.554 | DOHHUE |  |  |  |  |  |  |  |
| 63 | 2.554 | GEGBEB |  |  |  |  |  |  |  |
| 63 | 2.555 | FAWWOP |  |  |  |  |  |  |  |
| 63 | 2.555 | VEHBAN |  |  |  |  |  |  |  |
| 63 | 2.556 | XOSTUV |  |  |  |  |  |  |  |
| 63 | 2.580 | JOSTEQ01 |  |  |  |  |  |  |  |
| 63 | 2.581 | JOSTEQ |  |  |  |  |  |  |  |
|  |  |  |  |  |  | 63 | 2.620 | QOMNIP | close bidentate, +2 |
|  |  |  |  |  |  | 63 | 2.653 | RACWEA | anionic ligand |
|  |  |  |  |  |  | 63 | 2.692 | LUPHAH | +2 |
|  |  |  |  |  |  | 63 | 2.720 | RACWAW | +2 |
|  |  |  |  |  |  | 63 | 2.734 | XEPLEJ | +2 |
| 64 | 2.415 | GAWBEL |  |  |  |  |  |  | constrained |
| 64 | 2.425 | OBUJIE |  |  |  |  |  |  | constrained |
| 64 | 2.429 | VEDZOU |  |  |  |  |  |  | constrained |
| 64 | 2.436 | NAKYAZ |  |  |  |  |  |  | constrained |
| 64 | 2.443 | EXIMUT |  |  |  |  |  |  | close bidentate |
| 64 | 2.445 | YEGTOT |  |  |  |  |  |  | constrained |
| 64 | 2.452 | ZAZQEW |  |  |  |  |  |  | constrained |
| 64 | 2.460 | JEKWOL |  |  |  |  |  |  | anionic ligand |
| 64 | 2.464 | UFADEK |  |  |  |  |  |  | constrained |
| 64 | 2.476 | GAFDOH |  |  |  |  |  |  | constrained |
| 64 | 2.489 | XAKXIS |  |  |  |  |  |  |  |
| 64 | 2.501 | AQERET |  |  |  |  |  |  |  |
| 64 | 2.505 | TANMUQ |  |  |  |  |  |  | constrained |
| 64 | 2.508 | MARSII |  |  |  |  |  |  | constrained |
| 64 | 2.516 | ERAHIO |  |  |  |  |  |  |  |
| 64 | 2.519 | ERAHOU |  |  |  |  |  |  |  |
| 64 | 2.532 | ATAJUC |  |  |  |  |  |  |  |
| 64 | 2.532 | FIQPAW01 |  |  |  |  |  |  | constrained |
| 64 | 2.535 | ZETTID |  |  |  |  |  |  |  |
| 64 | 2.537 | VEHBER |  |  |  |  |  |  |  |
| 64 | 2.538 | FIQPAW |  |  |  |  |  |  | constrained |
| 64 | 2.540 | BUHHUJ |  |  |  |  |  |  |  |
| 64 | 2.540 | DOHJIU |  |  |  |  |  |  |  |
| 64 | 2.540 | GAKLOV |  |  |  |  |  |  |  |
| 64 | 2.540 | SUCWOE |  |  |  |  |  |  |  |
| 64 | 2.541 | CEYXUA |  |  |  |  |  |  |  |
| 64 | 2.550 | GEGBOL |  |  |  |  |  |  |  |
| 65 | 2.399 | KEPCOZ |  |  |  |  |  |  | constrained |
| 65 | 2.409 | VUKYOQ |  |  |  |  |  |  | constrained |
| 65 | 2.412 | QOQPIX |  |  |  |  |  |  | constrained |
| 65 | 2.419 | LOGBEQ |  |  |  |  |  |  | constrained |
| 65 | 2.434 | JERGAP |  |  |  |  |  |  | constrained |
| 65 | 2.439 | XUGROH |  |  |  |  |  |  | constrained |
| 65 | 2.445 | ZACTOP |  |  |  |  |  |  | constrained |
| 65 | 2.446 | JEKWUR |  |  |  |  |  |  | anionic ligand |
| 65 | 2.451 | BASLEN |  |  |  |  |  |  | constrained |
| 65 | 2.455 | DEGDUQ |  |  |  |  |  |  | constrained |
| 65 | 2.466 | JUCPOO |  |  |  |  |  |  | constrained |
| 65 | 2.478 | ADOQOB |  |  |  |  |  |  | constrained |
| 65 | 2.480 | CAMXUL |  |  |  |  |  |  | constrained |
| 65 | 2.484 | GOTCOH |  |  |  |  |  |  | constrained |
| 65 | 2.485 | AJAVUE |  |  |  |  |  |  | constrained |
| 65 | 2.489 | MUNHEI |  |  |  |  |  |  | constrained |
| 65 | 2.511 | IJOSUX |  |  |  |  |  |  | constrained |
| 65 | 2.512 | SAZLIQ |  |  |  |  |  |  | constrained |
| 65 | 2.517 | XOSVOR |  |  |  |  |  |  |  |
| 65 | 2.518 | EMATOD |  |  |  |  |  |  |  |
| 65 | 2.520 | BUHJEV |  |  |  |  |  |  |  |
| 65 | 2.521 | ATAJIQ |  |  |  |  |  |  |  |
| 65 | 2.526 | XOSVUX |  |  |  |  |  |  |  |
| 65 | 2.529 | EWONAH |  |  |  |  |  |  | constrained |
| 65 | 2.530 | GEGPOZ01 |  |  |  |  |  |  | constrained |
| 65 | 2.532 | EWONEL |  |  |  |  |  |  | constrained |
| 65 | 2.532 | EWONIP |  |  |  |  |  |  | constrained |
| 65 | 2.534 | GEGPOZ02 |  |  |  |  |  |  | constrained |
| 65 | 2.535 | EWOMUA |  |  |  |  |  |  | constrained |
| 65 | 2.535 | UJODEE01 |  |  |  |  |  |  | constrained |
| 65 | 2.535 | GEGPOZ |  |  |  |  |  |  | constrained |
| 65 | 2.541 | UJODEE |  |  |  |  |  |  | constrained |
| 65 | 2.548 | OQOZAX |  |  |  |  |  |  | constrained |
| 65 | 2.579 | JOSXAQ/01 |  |  |  |  |  |  | Eu+3? |
| 66 | 2.393 | TIQNIS |  |  |  |  |  |  | constrained |
| 66 | 2.404 | VUKYUW |  |  |  |  |  |  | constrained |
| 66 | 2.407 | JOBMIY |  |  |  |  |  |  | constrained |
| 66 | 2.410 | JERGET |  |  |  |  |  |  | constrained |
| 66 | 2.412 | VEDZUA |  |  |  |  |  |  | constrained |
| 66 | 2.421 | ZOHJOX |  |  |  |  |  |  | constrained |
| 66 | 2.432 | BASLIR |  |  |  |  |  |  | constrained |
| 66 | 2.436 | JEKXAY |  |  |  |  |  |  | anionic ligand |
| 66 | 2.439 | ZOCNEM |  |  |  |  |  |  | constrained |
| 66 | 2.440 | VOWTIM |  |  |  |  |  |  | constrained |
| 66 | 2.441 | VOWTOS |  |  |  |  |  |  | constrained |
| 66 | 2.444 | ZOCMUB |  |  |  |  |  |  | constrained |
| 66 | 2.445 | ZOCNAI |  |  |  |  |  |  | constrained |
| 66 | 2.446 | MAVQIL |  |  |  |  |  |  | constrained |
| 66 | 2.446 | VOWTEI |  |  |  |  |  |  | constrained |
| 66 | 2.450 | JUCLUQ |  |  |  |  |  |  | constrained |
| 66 | 2.451 | RAXZIB |  |  |  |  |  |  | constrained |
| 66 | 2.452 | MAVQOR |  |  |  |  |  |  | constrained |
| 66 | 2.457 | XAKXUE |  |  |  |  |  |  |  |
| 66 | 2.460 | OFUGOM |  |  |  |  |  |  | constrained |
| 66 | 2.460 | RAXZOH |  |  |  |  |  |  | constrained |
| 66 | 2.467 | AJAVUE |  |  |  |  |  |  | constrained |
| 66 | 2.468 | ADOQUH |  |  |  |  |  |  | constrained |
| 66 | 2.477 | MIKSAC |  |  |  |  |  |  | constrained |
| 66 | 2.488 | HAJHEI |  |  |  |  |  |  | constrained |
| 66 | 2.502 | MIKSEG |  |  |  |  |  |  | constrained |
| 66 | 2.505 | EMATET |  |  |  |  |  |  |  |
| 66 | 2.506 | ATAJOW |  |  |  |  |  |  |  |
| 66 | 2.509 | BUHJAR |  |  |  |  |  |  |  |
| 66 | 2.513 | RAYCAW |  |  |  |  |  |  |  |
| 66 | 2.514 | LABCAU |  |  |  |  |  |  |  |
| 66 | 2.520 | MIKRUV |  |  |  |  |  |  | constrained |
| 66 | 2.524 | KOBZEI |  |  |  |  |  |  | constrained |
| 66 | 2.528 | KOBZOS |  |  |  |  |  |  | constrained |
| 66 | 2.538 | GAKLUB |  |  |  |  |  |  |  |
| 66 | 2.544 | QODZEP |  |  |  |  |  |  |  |
| 67 | 2.412 | ZOHKAK |  |  |  |  |  |  | constrained |
| 67 | 2.415 | NAKYED |  |  |  |  |  |  | constrained |
| 67 | 2.416 | OQENAA |  |  |  |  |  |  | constrained |
| 67 | 2.435 | BASLOX |  |  |  |  |  |  | constrained |
| 67 | 2.458 | LEFWAV |  |  |  |  |  |  |  |
| 67 | 2.461 | XACHAM |  |  |  |  |  |  | constrained |
| 67 | 2.490 | LABCEY |  |  |  |  |  |  |  |
| 67 | 2.495 | KOJYAL |  |  |  |  |  |  |  |
| 67 | 2.502 | HULQOW |  |  |  |  |  |  |  |
| 67 | 2.503 | BUHJIZ |  |  |  |  |  |  |  |
| 67 | 2.504 | GABJIF |  |  |  |  |  |  |  |
| 67 | 2.525 | ACEKOJ |  |  |  |  |  |  | constrained |
| 68 | 2.366 | DIHZEZ |  |  |  |  |  |  | close bidentate |
| 68 | 2.370 | EXIMAZ |  |  |  |  |  |  | close bidentate |
| 68 | 2.374 | DIJQIW |  |  |  |  |  |  | close bidentate |
| 68 | 2.376 | DIJQES |  |  |  |  |  |  | close bidentate |
| 68 | 2.379 | DIJBAZ |  |  |  |  |  |  | close bidentate |
| 68 | 2.392 | EXINAA |  |  |  |  |  |  | close bidentate |
| 68 | 2.397 | WADZOR |  |  |  |  |  |  | constrained |
| 68 | 2.400 | ZOHJUD |  |  |  |  |  |  | constrained |
| 68 | 2.405 | EQULIN |  |  |  |  |  |  | constrained |
| 68 | 2.405 | EQULIN02 |  |  |  |  |  |  | constrained |
| 68 | 2.410 | YUHSUP |  |  |  |  |  |  | constrained |
| 68 | 2.431 | DEVSUS |  |  |  |  |  |  | constrained |
| 68 | 2.437 | FITKEZ |  |  |  |  |  |  | constrained |
| 68 | 2.440 | BASLUD |  |  |  |  |  |  | constrained |
| 68 | 2.440 | GOTCUN |  |  |  |  |  |  | constrained |
| 68 | 2.441 | XAKXEO |  |  |  |  |  |  |  |
| 68 | 2.450 | LEFVUO |  |  |  |  |  |  |  |
| 68 | 2.479 | UGEGEU |  |  |  |  |  |  |  |
| 68 | 2.483 | BERTID |  |  |  |  |  |  |  |
| 68 | 2.491 | DOHJAM |  |  |  |  |  |  |  |
| 68 | 2.493 | ATAJEM |  |  |  |  |  |  |  |
| 68 | 2.516 | VEHBIV |  |  |  |  |  |  |  |
| 69 | 2.402 | YEGTUZ |  |  |  |  |  |  | constrained |
| 69 | 2.411 | KUBMUQ |  |  |  |  |  |  |  |
| 69 | 2.427 | BASMAK |  |  |  |  |  |  | constrained |
| 69 | 2.442 | LEFWID |  |  |  |  |  |  |  |
| 69 | 2.452 | EXAXIM |  |  |  |  |  |  | constrained |
| 69 | 2.473 | UGEGIY |  |  |  |  |  |  |  |
| 69 | 2.486 | LAKZUU |  |  |  |  |  |  |  |
| 69 | 2.487 | DOHJOA |  |  |  |  |  |  |  |
| 70 | 2.340 | IWARIH |  |  |  |  |  |  | close bidentate |
| 70 | 2.347 | KEDVAQ |  |  |  |  |  |  | close bidentate |
| 70 | 2.348 | EYUKIS |  |  |  |  |  |  | constrained |
| 70 | 2.358 | YIXCAK |  |  |  |  |  |  | close bidentate |
| 70 | 2.374 | EXINEE |  |  |  |  |  |  | close bidentate |
| 70 | 2.395 | GIWWIS |  |  |  |  |  |  | acetonitrile |
| 70 | 2.402 | QOMNUB |  |  |  |  |  |  | close bidentate |
| 70 | 2.407 | BASMEO |  |  |  |  |  |  | constrained |
| 70 | 2.422 | AQERIX01 |  |  |  |  |  |  |  |
| 70 | 2.422 | MUVGER |  |  |  |  |  |  | constrained |
| 70 | 2.426 | XAKXAK |  |  |  |  |  |  |  |
| 70 | 2.438 | ICOXIJ |  |  |  |  |  |  | constrained |
| 70 | 2.439 | AQERIX |  |  |  |  |  |  |  |
| 70 | 2.441 | GOTDAU |  |  |  |  |  |  | constrained |
| 70 | 2.444 | YIYVAE |  |  |  |  |  |  | constrained |
| 70 | 2.450 | TABDIK |  |  |  |  |  |  | constrained |
| 70 | 2.451 | WUGBUV |  |  |  |  |  |  | constrained |
| 70 | 2.451 | XEPLIN |  |  |  |  |  |  |  |
| 70 | 2.456 | RIQCEA |  |  |  |  |  |  |  |
| 70 | 2.461 | UGEGOE |  |  |  |  |  |  |  |
| 70 | 2.467 | DOHJEQ |  |  |  |  |  |  |  |
| 70 | 2.469 | BORVIN |  |  |  |  |  |  |  |
| 70 | 2.469 | ZESROE |  |  |  |  |  |  |  |
| 70 | 2.475 | BUHJOF |  |  |  |  |  |  |  |
| 70 | 2.475 | KIFPIZ |  |  |  |  |  |  |  |
| 70 | 2.487 | TAJMUO |  |  |  |  |  |  |  |
| 70 | 2.490 | TAJNAV |  |  |  |  |  |  |  |
|  |  |  |  |  |  | 70 | 2.545 | RAYCEA | +2/+3? |
|  |  |  |  |  |  | 70 | 2.556 | AJOWIF | acetonitrile, +2? |
|  |  |  |  |  |  | 70 | 2.610 | LUPJEN | +2 |
|  |  |  |  |  |  | 70 | 2.639 | POSLEP | +2 |
|  |  |  |  |  |  | 70 | 2.639 | 431438 | +2 |
|  |  |  |  |  |  | 70 | 2.646 | 431437 | +2 |
| 71 | 2.344 | EXIMED |  |  |  |  |  |  | close bidentate |
| 71 | 2.352 | DIHZID |  |  |  |  |  |  | close bidentate |
| 71 | 2.361 | WAVNIS |  |  |  |  |  |  | constrained |
| 71 | 2.367 | PIXGUA |  |  |  |  |  |  | constrained |
| 71 | 2.368 | DULZAL |  |  |  |  |  |  | constrained |
| 71 | 2.368 | FABZUG |  |  |  |  |  |  | constrained |
| 71 | 2.369 | FACBAP |  |  |  |  |  |  | constrained |
| 71 | 2.376 | ORADES |  |  |  |  |  |  | constrained |
| 71 | 2.379 | DICBUM |  |  |  |  |  |  | constrained |
| 71 | 2.383 | VODDIB |  |  |  |  |  |  | constrained |
| 71 | 2.384 | GAVZUY |  |  |  |  |  |  | constrained |
| 71 | 2.389 | FABZOA |  |  |  |  |  |  | constrained |
| 71 | 2.390 | QOMNEL |  |  |  |  |  |  | close bidentate |
| 71 | 2.398 | NAKYIH |  |  |  |  |  |  | constrained |
| 71 | 2.407 | FEFCAW |  |  |  |  |  |  | constrained |
| 71 | 2.416 | LARNID |  |  |  |  |  |  | constrained |
| 71 | 2.417 | UFADOU |  |  |  |  |  |  | constrained |
| 71 | 2.420 | FITKID |  |  |  |  |  |  | constrained |
| 71 | 2.433 | LORKIM |  |  |  |  |  |  | constrained |
| 71 | 2.434 | CILZUT |  |  |  |  |  |  |  |
| 71 | 2.434 | XEPLOT |  |  |  |  |  |  |  |
| 71 | 2.436 | XEPLUZ |  |  |  |  |  |  |  |
| 71 | 2.441 | SOPCEG |  |  |  |  |  |  | anionic ligand |
| 71 | 2.444 | MARSOO |  |  |  |  |  |  | constrained |
| 71 | 2.446 | OBIPUL |  |  |  |  |  |  | acetonitrile |
| 71 | 2.473 | HAZHIZ |  |  |  |  |  |  |  |
| 71 | 2.487 | LARGUH |  |  |  |  |  |  |  |
| 58.17 | 2.603 | XANYIU |  |  |  |  |  |  |  |
| 58.17 | 2.619 | ELUYOZ |  |  |  |  |  |  |  |
| 58.17 | 2.624 | TAYRIW |  |  |  |  |  |  | anionic ligands |
| 58.17 | 2.628 | IBILAI |  |  |  |  |  |  | anionic ligands |
| 58.17 | 2.634 | CAKLEI |  |  |  |  |  |  | anionic ligands |

**Table S2** All nine-coordinate trivalent (left column set) and divalent (right column set) *N*-donor lanthanoid and actinoid structures reported in Cambridge Structural Database (CSD). The structures are ordered after atomic number, *Z*, mean Ln-N bond distance, and CSD or ICSD retrieval code.

| Z | *d*(Ln-N) | CSD or ICSD code |  |  |  |
| --- | --- | --- | --- | --- | --- |
| 57 | 2,594 | YUWLOT |  |  |  |
| 57 | 2,621 | QOJDUP |  |  |  |
| 57 | 2,628 | GIWVUD |  |  |  |
| 57 | 2,639 | ZACWIM |  |  |  |
| 57 | 2,641 | ZACWOS |  |  |  |
| 57 | 2,643 | XONYUU |  |  |  |
| 57 | 2,647 | BELGOP |  |  |  |
| 57 | 2,648 | IDUDEQ |  |  |  |
| 57 | 2,659 | IVEXOW |  |  |  |
| 57 | 2,660 | PUWTIL |  |  |  |
| 57 | 2,667 | PIZWAY |  |  |  |
| 57 | 2,669 | ZUWFOM |  |  |  |
| 57 | 2,676 | XONXUT |  |  |  |
| 57 | 2,683 | OHUWIY |  |  |  |
| 57 | 2,684 | XAWWAT |  |  |  |
| 57 | 2,695 | ZAXMAO |  |  |  |
| 57 | 2,705 | EGOBAD |  |  |  |
| 57 | 2,706 | 84283 |  |  |  |
| 57 | 2,723 | ZACBIR |  |  |  |
| 58 | 2,611 | ZACWUY |  |  |  |
| 58 | 2,617 | TIDKOG |  |  |  |
| 58 | 2,618 | TIDKIA |  |  |  |
| 58 | 2,618 | XONZAB |  |  |  |
| 58 | 2,624 | XONZEF |  |  |  |
| 58 | 2,630 | BUQHUR |  |  |  |
| 58 | 2,645 | XAWWEX |  |  |  |
| 58 | 2,650 | XONYAA |  |  |  |
| 58 | 2,655 | XONYEE |  |  |  |
| 58 | 2,656 | REPKUT |  |  |  |
| 58 | 2,675 | HORKOP |  |  |  |
| 59 | 2,553 | OBUHOI |  |  |  |
| 59 | 2,591 | GIWWEO |  |  |  |
| 59 | 2,597 | YUWMAG |  |  |  |
| 59 | 2,598 | ISUNAM |  |  |  |
| 59 | 2,599 | ISUMUF |  |  |  |
| 59 | 2,600 | OBUHIC |  |  |  |
| 59 | 2,614 | IJOTUW |  |  |  |
| 59 | 2,620 | BELHEG |  |  |  |
| 59 | 2,625 | REPLAA |  |  |  |
| 59 | 2,626 | REPLEE |  |  |  |
| 59 | 2,627 | XAWWOH |  |  |  |
| 59 | 2,630 | BELHAC |  |  |  |
| 59 | 2,637 | WUWWOA |  |  |  |
| 59 | 2,639 | IJOVIM |  |  |  |
| 59 | 2,640 | JOLBAO |  |  |  |
| 59 | 2,646 | NIYHEJ |  |  |  |
| 59 | 2,661 | HORLAC |  |  |  |
| 59 | 2,663 | LOQXEV |  |  |  |
| 59 | 2,663 | PUQMEV |  |  |  |
| 59 | 2,668 | RUGNOW |  |  |  |
| 59 | 2,678 | YOQDAL |  |  |  |
| 60 | 2,567 | GAPFIN |  |  |  |
| 60 | 2,571 | ZACXAF |  |  |  |
| 60 | 2,572 | ZACWEI |  |  |  |
| 60 | 2,580 | SIDVUX |  |  |  |
| 60 | 2,588 | YUWMEK |  |  |  |
| 60 | 2,601 | IJOVAE |  |  |  |
| 60 | 2,604 | IJOVEI |  |  |  |
| 60 | 2,619 | IJOVOS |  |  |  |
| 60 | 2,622 | REPLII |  |  |  |
| 60 | 2,626 | EBOYAW |  |  |  |
| 60 | 2,626 | XONYII |  |  |  |
| 60 | 2,634 | NIYHIN |  |  |  |
| 60 | 2,653 | BILSIY |  |  |  |
| 60 | 2,653 | EMEFIM |  |  |  |
| 60 | 2,654 | EMEFEI |  |  |  |
| 60 | 2,655 | LOQXIZ |  |  |  |
| 60 | 2,667 | RUGNUC |  |  |  |
| 62 | 2,534 | GIWWAK |  |  |  |
| 62 | 2,573 | XESBEC |  |  |  |
| 62 | 2,582 | XAWWUN |  |  |  |
| 62 | 2,586 | NIYHOT |  |  |  |
| 62 | 2,601 | IVIYAO |  |  |  |
| 62 | 2,607 | YIMCED |  |  |  |
| 62 | 2,610 | QAPLID |  |  |  |
| 62 | 2,622 | YOQDEP |  |  |  |
| 62 | 2,625 | DUWYOK |  |  |  |
| 62 | 2,628 | DUWYIE |  |  |  |
| 62 | 2,629 | HUDCIU |  |  |  |
| 62 | 2,632 | FITSEH |  |  |  |
| 63 | 2,536 | CIGJIM |  |  |  |
| 63 | 2,536 | LITJOO |  |  |  |
| 63 | 2,538 | RUPZEJ |  |  |  |
| 63 | 2,539 | WAKMOM |  |  |  |
| 63 | 2,542 | KAHVEX |  |  |  |
| 63 | 2,547 | NAWBEU |  |  |  |
| 63 | 2,552 | NAXRIN |  |  |  |
| 63 | 2,553 | KIFKOZ02 |  |  |  |
| 63 | 2,554 | KIFKOZ |  |  |  |
| 63 | 2,556 | ONEQUV |  |  |  |
| 63 | 2,557 | YUWLUZ |  |  |  |
| 63 | 2,562 | RULMAO |  |  |  |
| 63 | 2,565 | KIFKOZ01 |  |  |  |
| 63 | 2,566 | ORUSEB |  |  |  |
| 63 | 2,567 | PIZWEC |  |  |  |
| 63 | 2,569 | KEDZUQ |  |  |  |
| 63 | 2,572 | LIQCIZ |  |  |  |
| 63 | 2,575 | TPYREU01 |  |  |  |
| 63 | 2,575 | XAWWIB |  |  |  |
| 63 | 2,578 | WECHOB |  |  |  |
| 63 | 2,581 | EFAYAM |  |  |  |
| 63 | 2,581 | XITZAC |  |  |  |
| 63 | 2,583 | UQINIS |  |  |  |
| 63 | 2,584 | UBIQEB |  |  |  |
| 63 | 2,585 | ERUBIC |  |  |  |
| 63 | 2,585 | XITTOJ |  |  |  |
| 63 | 2,587 | NAZYET |  |  |  |
| 63 | 2,592 | BELGUV |  |  |  |
| 63 | 2,593 | YITPIA |  |  |  |
| 63 | 2,594 | HANMOY |  |  |  |
| 63 | 2,594 | PASYAK |  |  |  |
| 63 | 2,594 | PASYAK10 |  |  |  |
| 63 | 2,596 | REPBUK |  |  |  |
| 63 | 2,607 | EGOBEH |  |  |  |
| 63 | 2,616 | DIBYAQ |  |  |  |
| 63 | 2,619 | PUQMIZ |  |  |  |
| 63 | 2,623 | ZAWGAH |  |  |  |
|  |  |  | 63 | 2,808 | WIDREH |
| 64 | 2,541 | NAXROT |  |  |  |
| 64 | 2,554 | ORUSUR |  |  |  |
| 64 | 2,558 | PUWTOR |  |  |  |
| 64 | 2,567 | LIQCOF |  |  |  |
| 64 | 2,590 | XETBII |  |  |  |
| 64 | 2,607 | KEZFEB |  |  |  |
| 64 | 2,610 | YOQDIT |  |  |  |
| 65 | 2,517 | WAKMUS |  |  |  |
| 65 | 2,522 | WAKNED |  |  |  |
| 65 | 2,538 | RULMES |  |  |  |
| 65 | 2,545 | PUWVAF |  |  |  |
| 65 | 2,580 | UKUGEN |  |  |  |
| 66 | 2,498 | TUTXIP |  |  |  |
| 66 | 2,529 | ORUROK |  |  |  |
| 66 | 2,551 | PUWTUX |  |  |  |
| 66 | 2,584 | YOQDOZ |  |  |  |
| 67 | 2,503 | NAXRUZ |  |  |  |
| 68 | 2,500 | GABFAT |  |  |  |
| 68 | 2,514 | ITODOM |  |  |  |
| 68 | 2,518 | SOMTAR |  |  |  |
| 68 | 2,543 | SOMTEV |  |  |  |
| 68 | 2,556 | UKUGOX |  |  |  |
| 69 | 2,466 | TUTXOV |  |  |  |
| 69 | 2,499 | XESBIG |  |  |  |
| 70 | 2,463 | XESBOM |  |  |  |
| 70 | 2,475 | XENSAM |  |  |  |
| 70 | 2,475 | XESBUS |  |  |  |
| 70 | 2,496 | ITODEC |  |  |  |
| 70 | 2,506 | UQINOY |  |  |  |
| 70 | 2,527 | REPCEV |  |  |  |
| 70 | 2,546 | REPCAR |  |  |  |
| 70 | 2,563 | PUQMOF |  |  |  |
| 71 | 2,503 | XAWXEY |  |  |  |
| 71 | 2,505 | WICDUJ |  |  |  |
| 71 | 2,507 | FUTCUT |  |  |  |
| 71 | 2,516 | HORKUV |  |  |  |
| 58,17 | 2,542 | TIDKUM |  |  |  |
| 58,17 | 2,629 | XONYOO |  |  |  |
